# Early crocodylomorph increases top tier predator diversity during rise of dinosaurs

**DOI:** 10.1038/srep09276

**Published:** 2015-03-19

**Authors:** Lindsay E. Zanno, Susan Drymala, Sterling J. Nesbitt, Vincent P. Schneider

**Affiliations:** 1 Research & Collections, North Carolina Museum of Natural Sciences, Raleigh, NC 27601, USA; 2 Department of Biological Sciences, North Carolina State University, Raleigh, NC 27607, USA; 3 Department of Geosciences, Virginia Polytechnic Institute and State University, Blacksburg, VA 24061, USA

## Abstract

Triassic predatory guild evolution reflects a period of ecological flux spurred by the catastrophic end-Permian mass extinction and terminating with the global ecological dominance of dinosaurs in the early Jurassic. In responding to this dynamic ecospace, terrestrial predator diversity attained new levels, prompting unique trophic webs with a seeming overabundance of carnivorous taxa and the evolution of entirely new predatory clades. Key among these was Crocodylomorpha, the largest living reptiles and only one of two archosaurian lineages that survive to the present day. In contrast to their existing role as top, semi-aquatic predators, the earliest crocodylomorphs were generally small-bodied, terrestrial faunivores, occupying subsidiary (meso) predator roles. Here we describe *Carnufex* *carolinensis* a new, unexpectedly large-bodied taxon with a slender and ornamented skull from the Carnian Pekin Formation (~231 Ma), representing one of the oldest and earliest diverging crocodylomorphs described to date. *Carnufex* bridges a problematic gap in the early evolution of pseudosuchians by spanning key transitions in bauplan evolution and body mass near the origin of Crocodylomorpha. With a skull length of >50 cm, the new taxon documents a rare instance of crocodylomorphs ascending to top-tier predator guilds in the equatorial regions of Pangea prior to the dominance of dinosaurs.

The composition of modern ecosystems can be traced to the dynamic recovery of life in the aftermath of the catastrophic end-Permian mass extinction^1^. During the Triassic Period, unstable biotic communities^2^ and morphing ecospace^3^ gave rise to extant vertebrate clades such as frogs, lizards, mammals, turtles, and dinosaurs^1^,^4^,^5^ and spurred seemingly unbalanced trophic assemblages dominated by an excess of predatory taxa^6^. In the Middle Triassic, terrestrial predator assemblages included the small-bodied mesopredators Gracilisuchidae^7^ and top tier predator guilds dominated by poposauroids and basal loricatans (“rauisuchians”)^8^. By the Late Triassic, subsidiary predator guilds had shifted composition to newly emerging clades such as ornithosuchids^9^, early dinosaurs^4^,^5^, and the oldest known crocodylomorphs^10^. Whereas apex predator guilds were thought to be more highly conserved, retaining Middle Triassic representatives and expanding to include large-bodied theropod dinosaurs and rauisuchids^8^.

Here we describe a new species of crocodylomorph from the Carnian Pekin Formation, North Carolina, USA, representing one of the oldest, largest, and most basal crocodylomorphs yet discovered. This new taxon adds to a growing body of evidence that Triassic crocodylomorphs were more diverse than previously appreciated, and that theropod dinosaurs and crocodylomorphs exploited a wide trophic reach^11^, occupying both subsidiary and top-tier predator roles in the Late Triassic.

## Results

### Systematic paleontology

Archosauria Cope, 1869. Pseudosuchia Zittel, 1887–1890. Crocodylomorpha Walker, 1968 sensu Nesbitt 2011. *Carnufex carolinensis* gen. et sp. nov.

### Etymology

*Carnufex* (Latin) butcher; *carolinensis*, in reference to the region of discovery.

### Holotype

**Figure 1:**
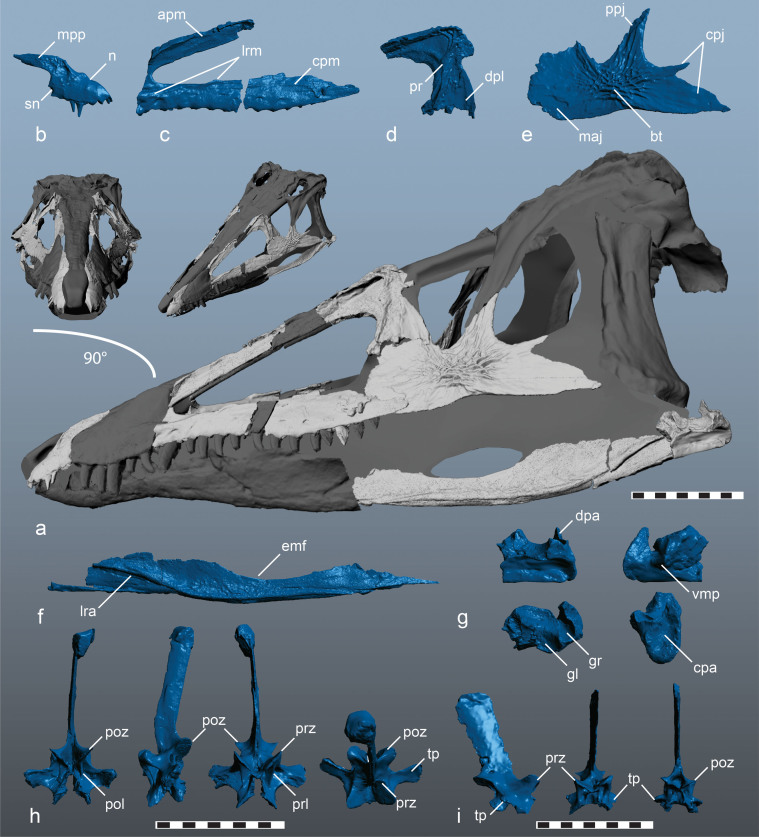
Three dimensional skull reconstruction and representative elements of *Carnufex carolinensis* (NCSM 21558). (a) reconstructed skull, clockwise from upper left, rostral, oblique, and lateral views; (b) right premaxilla, lateral view; (c) left maxilla, lateral view; (d) left lacrimal, lateral view; (e) left jugal, lateral view; (f) right angular, lateral view; (g) left articular, in (clockwise from upper left) lateral, medial, dorsal, caudal views; (h) cervical neural arch, in (left to right) caudal, lateral, cranial, and dorsal views; (i) dorsal neural arch, in (left to right) right lateral, cranial, and caudal views. Abbreviations: **apm**, ascending process, maxilla; **bt**, bulbous tuberosity; **cpa**, caudal process, articular; **cpj**, caudal process, jugal; **cpm**, caudal process, maxilla; **dpa**, dorsal process, articular; **dpl**, descending process, lacrimal; **emf**, external mandibular fenestra; **gl**, glenoid, **gr**, groove; **lra**, lateral ridge, angular; **lrm**, lateral ridge, maxilla; **maj**, maxillary articulation, jugal; **mpp**, maxillary process, premaxilla; **n**, naris; **pol**, centropostzygapophyseal lamina; **poz**, postzygapophysis; **ppj**, postorbital process, jugal; **pr**, prong; **prl**, centroprezygapophyseal lamina; **prz**, prezygapophysis; **sn**, subnarial notch; **tp**, transverse process; **vmp**, ventromedial process. Scale bar: 10 cm.

NCSM 21558, partial skull and postcranial skeleton including: right dentigerous premaxilla, left maxilla, left lacrimal, left jugal, left articular, right angular, isolated maxillary tooth, cervical neural arch, dorsal neural arch, cervical rib, dorsal ribs, and left humerus. (Fig. 1, S9).

### Referred materials

NCSM 21623, partial right humerus.

### Locality and horizon

Chatham County, North Carolina, USA; Pekin Formation, Chatham Group, Newark Supergroup, ~231 Ma^12^, Carnian, Late Triassic (SI–II).

#### Diagnosis

Large-bodied (~3 m) crocodylomorph distinguished by the following features (loricatan autapomorphies denoted by an asterisk): six premaxillary teeth*; horizontally directed maxillary process of premaxilla; elongate, subtriangular antorbital fenestra (length to height ratio ~2.3); caudodorsally trending lateral ridge on maxilla terminates at margin of antorbital fenestra*; caudal process of maxilla rostrally pinched, minimum dorsoventral height at rostralmost corner of antorbital fenestra*; jugal with ornamented lateral boss*; caudally deep antorbital fossa, with anteriorly directed flange extending from rostral margin of lacrimal*; caudal margin of antorbital fossa vertically oriented (caudodorsal corner directly dorsal to caudoventral corner)*; antorbital fossa more than twice the estimated area of the orbit*; bifurcated caudal process of jugal bearing a small caudodorsally directed flange*; small, sub-conical, medial process of articular; pronounced crainocaudally oriented ridge on caudal aspect of lateral surface of angular; ectepicondylar crest proximal to the radial condyle of the humerus.

#### Description

All neurocentral sutures remain open in NCSM 21558 indicating a skeletally immature individual^13^. The skull is rostrally elongate (estimated *minimum* length > 50 cm) and lightly built (Fig. 1a). The maxillary process of the premaxilla projects horizontally; it is subequal in length and parallel with, the alveolar margin (Fig. 1b). The premaxilla bears six premaxillary teeth (Fig. S9b), and a subnarial notch along the caudoventral margin of the tooth row (Fig. 1b, S1) as in *Dromicosuchus*^14^ NCSM 13733 (formerly UNC 15574), and the early crocodylomorph CM 29894. The palatal process exhibits a rostral palatal foramen (for the fourth dentary tooth^15^).

The antorbital fenestra is hypertrophied, circumscribed by a weakly developed antorbital fossa (Fig. 1c, S2) that contrasts with the expanded fossa and reduced fenestra of other loricatans and resembles that of poposauroids (e.g., *Arizonasaurus*) and early dinosaurs. The maxilla of *Carnufex* is transitional in possessing a strap-like, elongate ascending process at least 2/3rds the length of the antorbital fenestra. This is an intermediate condition between the craniocaudally reduced ascending process of other early diverging crocodylomorphs (e.g., *Dromicosuchus*^14^, *Sphenosuchus*^16^), and the relatively elongate, yet caudally expanded, ascending process of rauisuchids (e.g., *Polonosuchus*^17^).

A well-defined, rugose lateral ridge on the jugal process of the maxilla rises sharply to terminate at the ventral margin of the rostral antorbital fenestra (Fig. 1c), a condition otherwise undocumented in loricatans. A rugose, rostrally-oriented ridge on the rostral margin of the lacrimal bears a subtriangular prong, forming a keyhole shaped caudal margin of the antorbital fossa (Fig. 1d, S3). The descending process of the lacrimal widens rostrocaudally to contact the expanded rostral process of the jugal near the ventral orbital margin (Fig. 1a) as in crocodylomorphs. However, unlike crocodylomorphs, the ventral portion of the orbit in *Carnufex* is craniocaudally compressed, a condition that more closely resembles rauisuchids. The orbital area is markedly smaller than the antorbital fossa (<50%). The caudal process of the jugal is bifurcated (Fig. 1e, S4), a synapomorphy of Dinosauria, also present in *Proterosuchus*^18^.

The angular is slender and rims an elongate external mandibular fenestra (minimum 10 cm in length). In lateral view, the caudal aspect folds into a pronounced ridge (Fig. 1f. S5) as in *Junggarsuchus*^19^, likely representing the insertion point for the m. pterygoideus ventralis^20^. The articular bears a saddle-shaped glenoid, as in crocodylomorphs generally. A ventromedial process of the articular is present, yet reduced, differing from the tongue-like condition of other loricatans^21^. The caudodorsal surface of the caudal process of the articular is concave as in *Dromicosuchus*^14^ and *Protosuchus* *richardsoni* and bears a dorsomedial projection as in other basal crocodylomorphs^14^,^16^. In *Carnufex*, this projection is separated from the glenoid by a deep groove (Fig. 1g, S6) that is otherwise absent in crocodylomorphs more closely related to Crocodyliformes^18^.

Premaxillary tooth crowns are elongate, serrated, and slightly recurved, whereas the caudal maxillary tooth is serrated on mesial and distal carinae, stout and blade-like, with a weakly convex distal carina. All cranial elements except the articular are ornamented. Anastomosing pits and grooves are most pronounced on the jugal, where they form a rounded tuberosity (Fig. 1e), and on the lacrimal, where they coalesce into a rugose crest on the caudodorsal margin of the antorbital fossa (Fig. 1d). The large bodied, crocodylomorph *Redondavenator* also exhibits pronounced cranial ornamentation^11^, as do some large-bodied rauisuchids (e.g., *Postosuchus*^22^). Ornamentation is weak to absent in small-bodied basal crocodylomorphs (e.g., *Sphenosuchus*^16^, *Dromicosuchus*^14^) suggesting a possible correlation with body size.

The cervical neural arch (Fig. 1h, S7) exhibits dorsoventrally expanded diapophyses; and transversely elongate, steeply inclined pre- and postzygapophyses. Nine bilateral laminae support the neural arch, framing infrapre- and infrapostzygapophyseal fossae and centrodiapophyseal fossae, with accessory divisions. A single dorsal neural arch possesses centroprezygapophyseal fossae and diminutive, pendant transverse processes (Fig. 1i, S8). The humerus is relatively short (<45% of estimated skull length), with a transversely expanded distal end that is proportionally consistent with rauisuchids (~300% shaft width), as opposed to crocodylomorphs (<200%). An ectepicondylar groove and a supinator process are present, as in aetosaurs, other loricatans, yet in contrast to other crocodylomorphs.

## Discussion

**Figure 2:**
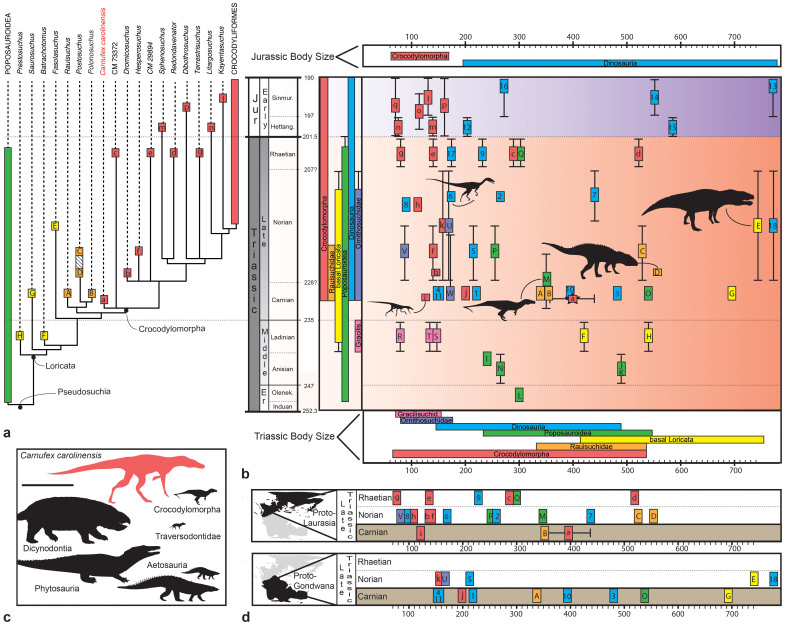
Evolutionary relationships, chronostratigraphic distribution, and estimated body size for putatively terrestrial, carnivorous archosaurians of the Triassic and earliest Jurassic. (a) Chronostratigraphically calibrated strict consensus tree showing taxonomy and relationships of the pseudosuchian clade Loricata. *Carnufex carolinensis* posited as a basalmost crocodylomorph. (b) Chronostratigraphically calibrated, bivariate plots of body size in terrestrial carnivorous archosaur clades with clade-specific temporal distributions summarized on the y-axis, and Triassic and earliest Jurassic body size ranges summarized on lower and upper x-axes, respectively. (c) Body size distribution in the Triassic divided by proto-Laurasia and proto-Gondwana. (d) Tetrapod composition of the Carnian-aged Pekin Formation (~231 Ma) to scale. Size estimates based on the proxy femur length (FL) in mm. Error bars denote stratigraphic and FL uncertainty. Key for taxon symbols a-q; A-V; 1–18 in Table S2. Taxon colors consistent between subparts. Paleomaps produced in Adobe Photoshop CS5.1. Scale bar in subpart (c) = 1 m.

A comprehensive phylogenetic analysis of Archosauria including 79 taxa and 413 characters^7^ posits *Carnufex* at the base of Crocodylomorpha in an unresolved polytomy with the skeletally immature postcranial skeleton CM 73372 (Fig. 2a, S9). We also provide the first phylogenetic placement of the Rhaetian pseudosuchian *Redondavenator*, substantiating this taxon as the largest Triassic crocodylomorph yet described^11^ (Fig. S9). *Carnufex* and *Redondavenator* expand the diversity of top tier terrestrial predator guilds in the Late Triassic to at least five distinct archosaur clades and document vast overlap in body size between contemporary dinosaurs and crocodylomorphs (Fig. 2b). In contrast, the Triassic-Jurassic transition marks a shift to dichotomous body mass distributions between terrestrial members of these two clades, and a loss of top tier crocodylomorph diversity after the end-Triassic extinction (ETE) (Fig. 2b).

The Carnian-aged Pekin Formation preserves some of the oldest Triassic archosaur assemblages in North America and brings to bear unique biodiversity data on the composition of top predator guilds preceding the appearance of theropod dinosaurs on the continent^18^,^23^. To date, tetrapods of the Pekin Formation are well sampled, and capture a diverse assemblage comprised of dicynodontians^24^, traversodontid cynodontians^25^, aetosaurians^26^, and two species of crocodylomorphs (*Carnufex*, and a new small bodied taxon^27^ with a femur length {**FL**} = 133 mm) (Fig. 2c). With an estimated immature FL of 353–440 mm, *Carnufex* is the largest terrestrial predator in the Pekin Formation (Fig. 2d), vastly exceeding the body size of the earliest North American theropod dinosaurs (FL 174–265 mm) (Fig. 2b).

Early concepts of faunal homogenization across Pangea are unsupported by recent studies, which instead document latitudinally arrayed, paleoclimatic faunal provinces across the supercontinent^12^,^28^, although this pattern is likely restricted to assemblages preceding the ETE^29^. Predatory guild evolution in the Triassic was equally complex, with recent research supporting a diachronous replacement of the leading terrestrial predators—pseudosuchians—with theropod dinosaurs between proto-Laurasian and Gondwanan landmasses^23^,^30^. Our chronostratigraphic plots of body size (Fig. 2b) and paleogeographic region (Fig. 2d) support both hypotheses. Whereas Carnian terrestrial predator guilds in southern hemisphere faunas were exploited in part by large bodied theropod dinosaurs, northern and equatorial faunas of similar age have yet to yield definitive theropod remains^5^,^23^,^30^ and appear instead to have been evolutionary centers for large bodied terrestrial crocodylomorphs^11^, as exemplified by *Carnufex* in the Carnian and subsequently by *Redondavenator* in the Rhaetian. The loss of large-bodied crocodylomorphs nearing the ETE may have spurred mesopredator release^31^ or opportunistic invasion scenarios^30^, whereby smaller-bodied theropods subsequently assumed apex predator roles in paleoequatorial regions of proto-Laurasia.

## Methods

### Body size

We evaluated body size using the widely accepted proxy of femur length (FL)^32^,^33^,^34^. Measurements of FL were derived primarily from recent archosaurian datasets^32^,^35^. Select taxa relevant to our analyses lack a femur and required FL estimation. We derived scaling equation (1) for predicting FL by applying OLS regression analysis on bivariate plots of FL/humeral length (HL) (Fig. S10) and equation (2) using FL/skull length (SKL) (Fig. S11) for a variety of loricatans for which data were available (Table S1). We also performed OLS regressions on bivariate plots of log e HL against SKL (Fig. S12) to examine the reliability of our estimates against independent scaling relationships (equation [3]). Data was log transformed (log e). Scaling equations had high coefficients of determination (R^2^) ranging from 0.94–0.97; however, these are influenced by low sample size (n = 12)^36^.
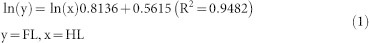

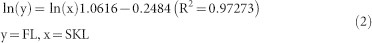

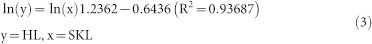


We used scaling equations to estimate FL of *Trialestes* *romeri* based on a 220 mm measurement of SKL provided by Reig^37^; *Redondavenator quayensis* based on a 600 mm SKL estimate provided by Nesbitt et al.^11^; *Pseudhesperosuchus* *jachaleri* based on an estimated skull length of 130 mm^38^, *Dibrothosuchus* *elaphros* based on 112.5 mm HL and 164 mm SKL^15^, *Phyllodontosuchus lufengensis* based on a 71.5 mm SKL^39^, *Pedeticosaurus* *leviseuri* based on a HL of 82 mm^40^, *Yonghesuchus* *sangbiensis* based on a 155 mm SKL^7^. We used comparative SKL to FL measurements provided by Sues et al.^41^ and Smith et al.^42^ to approximate the FL of *Daemonosaurus* *chauliodus* and *Zupaysaurus rougieri*. At 145 mm, the SKL of *D. chauliodus* closely approximates the estimated SKL of *Tawa* *hallae* (147 mm^41^), whereas, the estimated SKL of *Z*. *rougieri* closely approximates that of *Cryolophosaurus* *ellioti* (FL 769 mm^42^). We did not estimate FL of the Early Jurassic theropods *Dracovenator* *regenti* and *Lophostropheus airelensis* because of the fragmentary nature of the remains (all lack appendicular elements and complete skulls) and because our conclusions revolve around Late Triassic fauna in the former instance. However, we note that published estimates of *D*. *regenti* size (5.5–6.5 m in length^42^) would place this taxon with the range of body size already captured in Figure 2, adding no new data to our results. Estimates of FL generated for these taxa are listed in Table S2 and marked with an asterisk. We only provide a general estimate of FL for the ornithosuchid *Venaticosuchus* as approximating that of the ornithosuchid *Riojasuchus*, because the skull of this taxon is highly fragmentary and appears to have been close in size to *Riojasuchus*^9^.

We derived a FL range for *Carnufex* *carolinensis* of 354–441 mm, based on a measured HL of 207.7 mm and estimated *minimum* SKL of 500 mm, respectively. Given the large FL range produced from these two variables, we further explored FL for *Carnufex* by testing how accurately we could approximate known HL and estimated SKL values using the relevant scaling equation derived from our loricatan dataset. Our estimation of *Carnufex* HL using a minimum SKL length of 500 mm was 257 mm, 24% larger than our actual measurement on the preserved humerus of NCSM 21558. Conversely, our estimate of *Carnufex* SKL using the actual value for HL of CNSM 21558 was 385 mm, far shorter than the portion of the skull preserved (450 mm). These data indicate that the humeral to skull proportions of *Carnufex* are not a good fit to the regression, i.e., either the humerus of *Carnufex* is unusually short, or the skull unusually long, or both variations are compounded. Therefore, we present FL estimates derived both from the HL and SKL here. We note that NCSM 21558 is skeletally immature, having open neurocentral sutures across the cervical and dorsal series minimally. This immaturity plus our use of a minimum estimate for skull length yields a conservative range of 354–441 mm for FL. We expect the FL of a somatically mature *Carnufex* would fall within the upper values of our current estimates or perhaps well above. Our gross estimate of the body length of a skeletally immature *Carnufex* (~3 m) is based on comparative skeletal ratios in the closely related *Dromicosuchus* (NCSM 13731) and the nearly complete basal crocodylomorph NCSM 21722^27^.

### Ecological inferences

Given that autecology cannot be observed for extinct taxa, paleontologists generally rely on the presence of ecomorphological traits to infer dietary inferences^43^,^44^,^45^,^46^ and construct trophic networks^2^,^48^,^49^. We follow Mitchell & Makovicky^49^ in assigning the extinct archosaurs to guilds (e.g., top-tier predator) based on body size, inferred diet, and habitat (e.g., terrestrial, semi-aquatic, aquatic). These ecological factors were taken from the published literature. We followed multiple authors in considering taxa of the following clades to represent the diversity of carnivorous, terrestrial, Triassic pseudosuchians: loricatans (Rauisuchidae + Crocodylomorpha)^6^,^10^, gracilisuchids^7^, poposauroids^50^, & ornithosuchids^9^; and in assigning early theropods to this guild^5^. Dietary inferences for some Triassic pseudosuchians are ambiguous (e.g., *Effigia*^51^); however, without quantitative analyses testing analogous ecomorphological traits in these taxa^45^,^47^, we include them as carnivores in keeping with the apparent dominant trophic habit of their clade, considering this a conservative approach. Currently known diversity places *Carnufex* and *Redondavenator* as the largest, terrestrial carnivores within their respective assemblages^11^, which generally denotes apex predator status^30^. However, given the potential of sampling biases (e.g., no rauishuchids recovered from the Pekin Formation) and the nuances of extant predator interaction^6^,^30^, we refrain from restricting these taxa to apex predator roles. Rather, we adopt a more conservative approach that allows for incomplete sampling of large-bodied carnivores, by considering *Carnufex* and *Redondavenator* to be minimally, components of top-tier predator guilds within Triassic faunas.

### Phylogenetic protocol

We examined the evolutionary relationships of *Carnufex* and *Redondavenator* by inclusion in the recent, comprehensive analysis of archosaurs published by Butler et al.^7^, which is an expansion of Nesbitt^18^. The analysis includes 79 archosaurs and 413 characters. We followed Butler et al.^7^ in a priori exclusion of the operational taxonomic units: *Archosaurus* *rossicus*, *Prestosuchus* *chiniquensis*, UFRGS 0156 T, UFRGS 0152 T, *Lewisuchus* *admixtus*, and *Pseudolagosuchus* *major*; and in designation of the following characters as additive: 32, 52, 75, 121, 137, 139, 156, 168, 188, 223, 247, 258, 269, 271, 291, 297, 328, 356, 399, and 413. Data coding, character tracing and tree manipulation/visualization were carried out using Mesquite ver. 2.75^52^. Phylogenetic analyses were executed in the program TNT^53^. We conducted heuristic searches on Wagner trees using TBR (tree bisection–reconnection) branch-swapping with 1,000 random addition sequences holding 10 trees per replicate, continuing subsequent TBR swapping on all stored minimum length trees (90 most parsimonious trees, TL 1,320). We assessed results using strict and reduced consensus methods and Bremer support values^54^. Ambiguous nodes were collapsed following Rule 1 of Coddington and Scharff^55^. Maximum agreement subtrees^56^ were calculated in TNT and used to identify labile taxa and common topology among all MPTs.

In this analysis we recover three unambiguous synapomorphies of Crocodylomorpha + *Carnufex*: a sub narial gap (char. 11 state 1); an elongate lacrimal reaching the ventral aspect of the orbit (char. 39, state 1); and loss of fin-like hyposphen-hypantrum articulations in the vertebral series (char. 195, state 0). However, *Carnufex* clearly exhibits a mosaic bauplan that spans lightly built, cursorial crocodylomorphs and their large-bodied, robust sister taxa, rauisuchids. As a result, *Carnufex* also shares several skeletal features characteristic of rauisuchids including a bulbous longitudinal ridge on the maxilla (char. 26, state 2); non-tapering dorsal process of the maxilla (char. 29, state 1); as well as retaining some synapomorphies of Loricata, lost in crocodylomorphs more closely related to *Alligator* than *Carnufex*, including a distinct groove caudal to the glenoid fossa on the articular (char. 156, state 1); and a tall, narrow orbit (char. 142, state 1). *Carnufex* also possesses some traits convergent with theropod dinosaurs such as a bifurcated caudal process of the jugal (char. 71, state 3) and a dorsoventrally expanded caudal process of the jugal, also present in *Revueltosaurus* and some archosauromorphs (char. 27, state 2). We recover *Carnufex* as an unequivocal crocodylomorph in our analysis. Two steps are required to move *Carnufex* out of Crocodylomorpha (Fig. S9). The remaining nodes within Crocodylomorpha are supported by Bremer values of 1 (Fig. S9). The mosaic morphology exemplified by this taxon and its basal phylogenetic and stratigraphic position yields critical insight into the step-wise appearance of the crocodylomorph bauplan.

*Redondavenator* *quayensis* was described as a large bodied crocodylomorph^11^, yet has not been tested in a phylogenetic context. Although fragmentary (only 8% of characters can be coded) we included this taxon to substantiate this placement quantitatively. Our analysis posits *R. quayensis* as sister-taxon to *Sphenosuchus* *acutus* based on the shared presence of an elongated maxillary process of the premaxilla (char 2, state 1). Although the maxillary process of the premaxilla is incomplete in *Redondavenator*, we find that all taxa possessing five or more maxillary alveoli anterior to the antorbital fenestra also possess a maxilla in which the portion rostral to the antorbital fenestra is longer than the posterior process (char 2, state 1) (SD per obs.). This correlation may not prove exhaustive, given that *Redondavenator* only includes the anterior portion of the skull; however, we include coded *Redondavenator* for this trait as a testable hypothesis.

The skeletally immature postcranial skeleton CM 73372 has been variously interpreted as *Postosuchus*^28^,^57^,^58^ and a *Hesperosuchus*-like basal crocodylomorph^18^. Our analysis is unable to resolve the relationship between *Carnufex* and CM 73372, recovering these taxa in a polytomy with a clade consisting of all remaining crocodylomorphs. *Carnufex* is represented predominantly by cranial elements and CM 73372 consists entirely of postcranial elements, therefore there is little overlapping data between these species to aid in phylogenetic resolution.

### 3D visualization and reconstruction

Elements of the skull and postcranial skeleton of *Carnufex* were scanned using a Creaform EXAscan^TM^ high-resolution (0.050 mm) handheld surface scanner. Scans were captured in 1.0–0.02 mm resolution using VXelements 3D data acquisition software. Post processing and generation of 3D PDFs were accomplished in Geomagic Studio®. We used Autodesk Maya 2014 to produce a composite three-dimensional model of the skull using individual element scans of *Carnufex* (NCSM 21623), supplemented with scaled surface scans of cranial elements of the closely related crocodylomorph *Dromicosuchus*, and *Junggarsuchus*, performed using the same protocol as *Carnufex*. Skull width was modeled using the relatively undeformed skull roof of *Dromicosuchus*. The rostral dentary and braincase were modeled using scans of *Junggarsuchus*, whereas the rostral maxilla, quadrate, frontals, parietals, squamosals, nasals, and maxillary dentition were modeled from *Dromicosuchus*. The circumnarial region of the premaxilla, prefrontal, quadratojugal and remainder of the mandible were generated as de novo objects manipulated to reflect estimated proportions. Original three-dimensional scans of the skeletal elements of *Carnufex* are provided as individual 3D PDFs as Supplementary Information (Figs. S1–8).

### Methods summary

This published work and the nomenclatural acts it contains have been registered in ZooBank, the proposed online registration system for the International Code of Zoological Nomenclature (ICZN). The ZooBank LSIDs (Life Science Identifiers) can be resolved and the associated information viewed through any standard web browser by appending the LSID to the prefix ‘http://zoobank.org/'. The LSID for this publication is: urn:lsid:zoobank.org:pub:129E5681-0D2E-4A7D-BC04-A3E624151BF7.

## Author Contributions

L.Z. and S.D. jointly conceived the project, described fossil materials, analyzed the data, and wrote the manuscript with contributions from S.N and V.S. L.Z., S.D. and S.N. coded the specimen for phylogenetic analysis; L.Z. performed phylogenetic analyses; V.S. collected fossil materials.

### Additional information

Phylogenetic Data archived as Project 2125 at Morphobank (www.morphobank.org).

Reprints and permissions information is available at www.nature.com/reprints

## Supplementary Material

Supplementary Information Main Supplementary File

Supplementary Information Figure S1

Supplementary Information Figure S2

Supplementary Information Figure S3

Supplementary Information Figure S4

Supplementary Information Figure S5

Supplementary Information Figure S6

Supplementary Information Figure S7

Supplementary Information Figure S8
