# Supplementary material for: Early crocodylomorph increases top tier predator diversity during rise of dinosaurs
Source: Sci Rep. 2015 Mar 19;5:9276. doi: 10.1038/srep09276 (PMC4365386; doi:10.1038/srep09276)
Supplement: Supplementary Information — Main Supplementary File [file srep09276-s1.docx]

Supplementary Information for:

**Early crocodylomorph increases top predator diversity during rise of dinosaurs**

**Lindsay E. Zanno^1,2*^, Susan Drymala^1,2*^, Sterling J. Nesbitt^3^, & Vincent P. Schneider^1^**

^1^Research & Collections, North Carolina Museum of Natural Sciences, Raleigh, NC 27601, USA

^2^Department of Biological Sciences, North Carolina State University, Raleigh, NC 27607, USA

^3^Department of Geosciences, Virginia Polytechnic Institute and State University, Blacksburg, VA 24061, USA

CONTENTS

I. Stratigraphic Information

II. Character codes for *Carnufex* & *Redondavenator*

III. References #59-#61

Figure S1: 3D PDF of right premaxilla*

Figure S2: 3D PDF of left maxilla*

Figure S3: 3D PDF of left lacrimal*

Figure S4: 3D PDF of left jugal*

Figure S5: 3D PDF of right angular (reversed)*

Figure S6: 3D PDF of left articular*

Figure S7: 3D PDF of cervical neural arch*

Figure S8: 3D PDF of dorsal neural arch*

Figure S9: Photographic figure of NCSM 21623

Figure S10: Strict consensus tree with support values

Figure S11: OLS linear regression of humeral length against femur length in Loricata

Figure S12: OLS linear regression of skull length against femur length in Loricata

Figure S13: OLS linear regression of skull length against humeral length in Loricata

Table S1: Taxonomic dataset for scaling equations

Table S2: Femur length of carnivorous pseudosuchians used in bivariate plots

of body mass and geologic time (Fig. 2B,C)

Table S3: Select measurements of *Carnufex* *carolinensis*

*3-dimentional PDFs of individual elements included as separate files.

**I. Stratigraphic Information**

The Deep River Basin of North and South Carolina represents the southernmost exposure of Upper Triassic sequences deposited on the east coast of North America during the rifting of Pangea^59^. The basin is further divided along a northeast/southwest transect into the Wadesboro, Sanford, and Durham nominal subbasins. The Pekin Formation is the oldest strata in the Chatham Group, overlain by the Cumnock and Sanford formations respectively^60^. *Carnufex* *carolinensis* was recovered from an active brick quarry in the mid-upper Pekin Formation, northern Sanford Basin (detailed locality data on file at NCMNS, locality NCPALEO 1902). The specimen was discovered in a boulder field out of stratigraphic context. Its exact stratigraphic position within Pekin Fm. has not yet been determined; however, the lithology of these boulders is consistent with a channel and/or alluvial fan deposit located near the base of the quarry^60^. Paleomagnetic data indicates that the Pekin Formation was deposited 2°S of the Triassic paleoequator at ~231 Ma^12^. The overlying Cumnock Formation, from which *Postosuchus* *alisonae* and *Dromicosuchus* *grallator* were recovered, was deposited at the equator between 226-224 Myr^12^.

In dating to 231 Ma, *Carnufex* is older than or penecontemporaneous with oldest confirmed crocodylomorph *Trialestes romeri*, which stems from lower to middle strata of the Ischigualasto Formation (231-227 Myr^61^). At least two specimens of *Trialestes* are known. Although the holotype derives from the Cancha de Bochas Member (lower third of the formation [231-228 Myr^61^) a specimen referred to *Trialestes* *romeri* was recovered near Cerro Las Lajas^10,62^, which may preserve younger deposits (227 Ma)^10^, although these beds have not been dated as other part of the Ischigualasto Formation. Based on this association, a reasonable estimate for *Trialestes* would be closer to 228 Ma^10^; however, it is not yet clear if the two specimens represent the same taxon.

The Pekin Formation preserves an entirely new window on equatorial Pangean assemblages. Although the vertebrate fauna remains mostly undescribed, a diversity of specimens have been recovered, almost all of which represent novel taxa. These include: a new aetosaur^26^; two new crocodylomorphs (*Carnufex* and a small bodied taxon^27^; a traversodont cynodont formerly assigned to *Boreogomphodon*^25^; and a new dicynodont^24^.

**II. Character codes for *Carnufex* & *Redondovenator***

*Carnufex carolinensis*

?00?0300001?0010000?0000022?11???????01?????????????????????????0?00113?000????????????????????????????????????????????????????????????12100?1?????????????11?1??0?1??01000001????????????0??????00?00???????????????????????????????0???000????????????????????????????????????????????????????????????????????????????????????????????????????????????????????????????????????????????????????????????????????????????????0

*Redondovenator quayensis*

010?0200001001???000?0?00??????2??0???????????????????????????????????????????????????????????????????????????????????????????????????????00??????????????????????????01000001????????????????????????????????????????????0??11??????????????????????????????????????????????????????????????????????????????????????????????????????????????????????????????????????????????????????????????????????????????????????????????

1. **References #59-62**

59. Olsen, P. E. & Huber, P. The oldest Late Triassic footprint assemblage from North America (Pekin Formation, Deep River basin, North Carolina, USA). *Southeastern Geol.* **38**, 77–90 (1998).

60. Heckert, A. B., Schneider, V. P., Fraser, N. C., Webb, R. A. A new aetosaur (Archosauria, Suchia) from the Upper Triassic Pekin Formation, Deep River Basin, North Carolina, U.S.A. and its implications for early aetosaur evolution. *J. Vertebr. Paleontol.* (in press).

61. Martinez, R. N. et al. A basal dinosaur from the dawn of the dinosaur era in south-western Pangea. *Science* **331**, 206–210 (2011).

62. Bonaparte, J. F. El Mesozoico de Americá del Sur y sus tetrapodos. *Opera* *Lilloana*, **26**, 1–596 (1978).


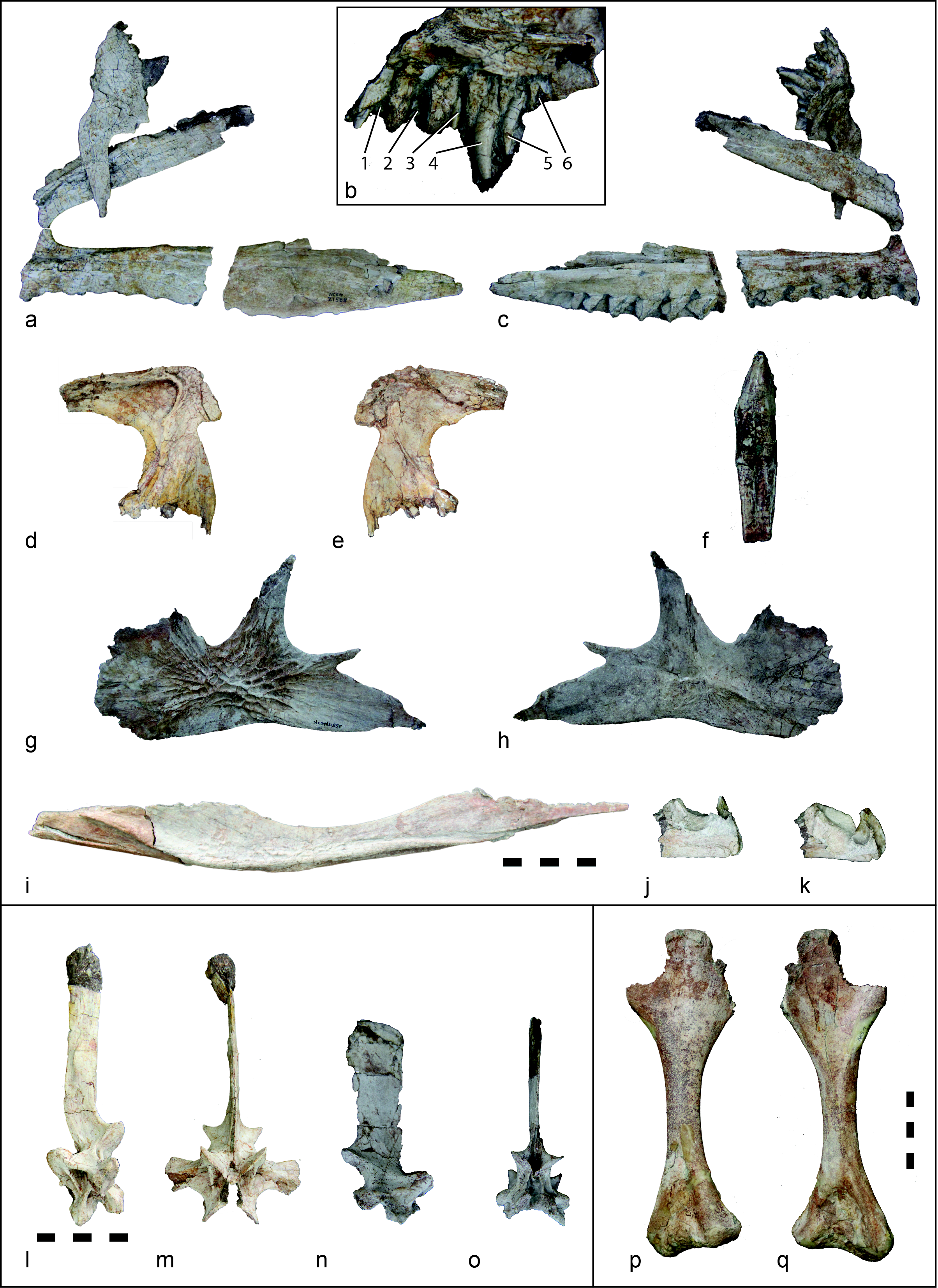


Figure S9. Photographic figure of NCSM 21623). right premaxilla and maxilla in lateral (**a**) and medial (**c**) views; (**b**) right premaxilla detail of teeth and alveoli (not to scale); left lacrimal, lateral (**d**) and medial (**e**) views; (**f**) isolated maxillary tooth with root; left jugal in lateral (**g**) and medial (**h**) views; (**i**) right angular, lateral view; left articular in lateral (**j**) and medial (**k**) views; cervical neural arch in left lateral (**l**) and cranial (**m**) views; dorsal neural arch in right lateral (**n**) and caudal (**o**) views; right humerus in caudal (**p**) and cranial (**q**) views. Scale bar corresponds to all elements in boxes: 5 cm.


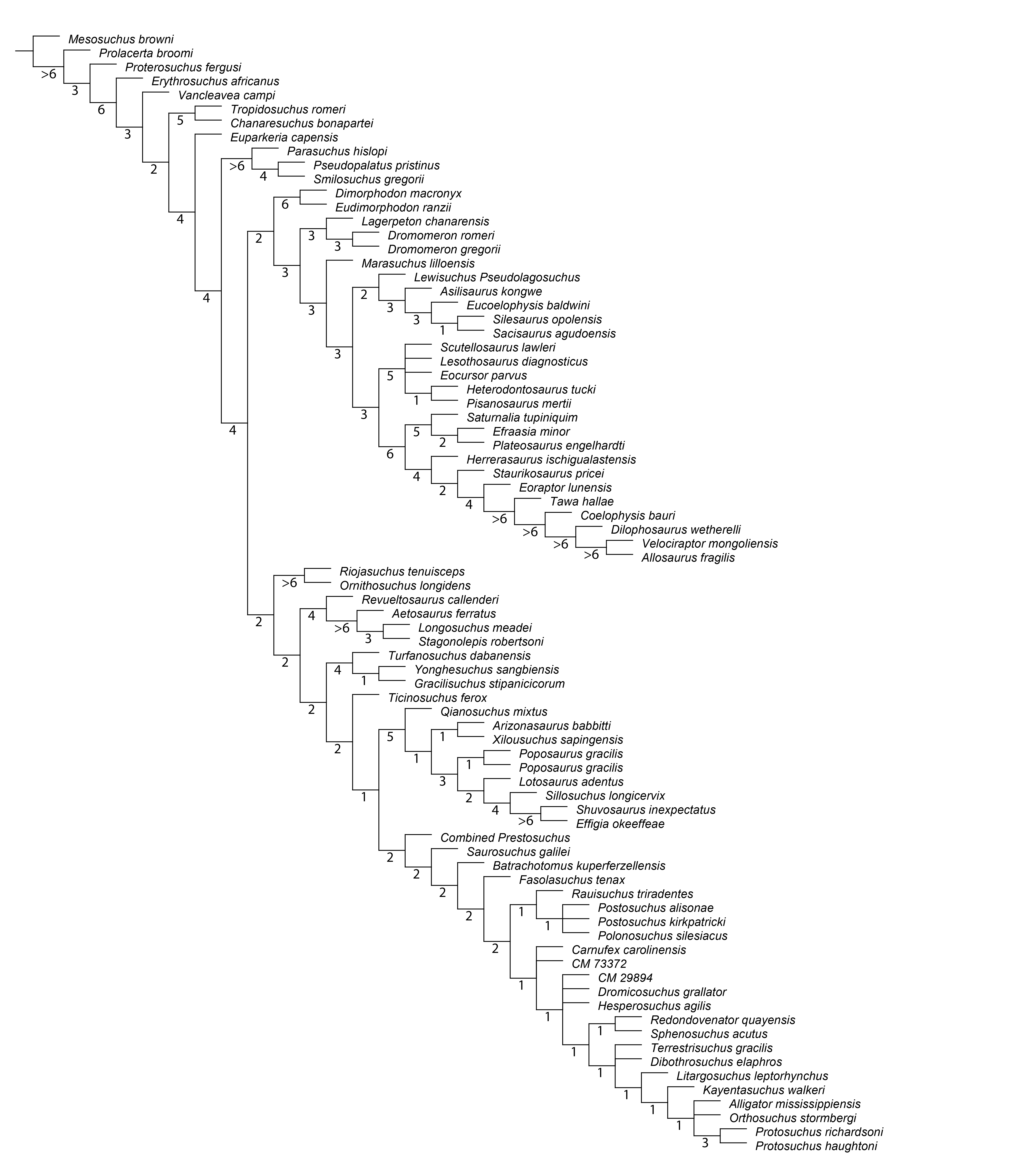


Figure S10. Strict consensus tree with Bremer values.


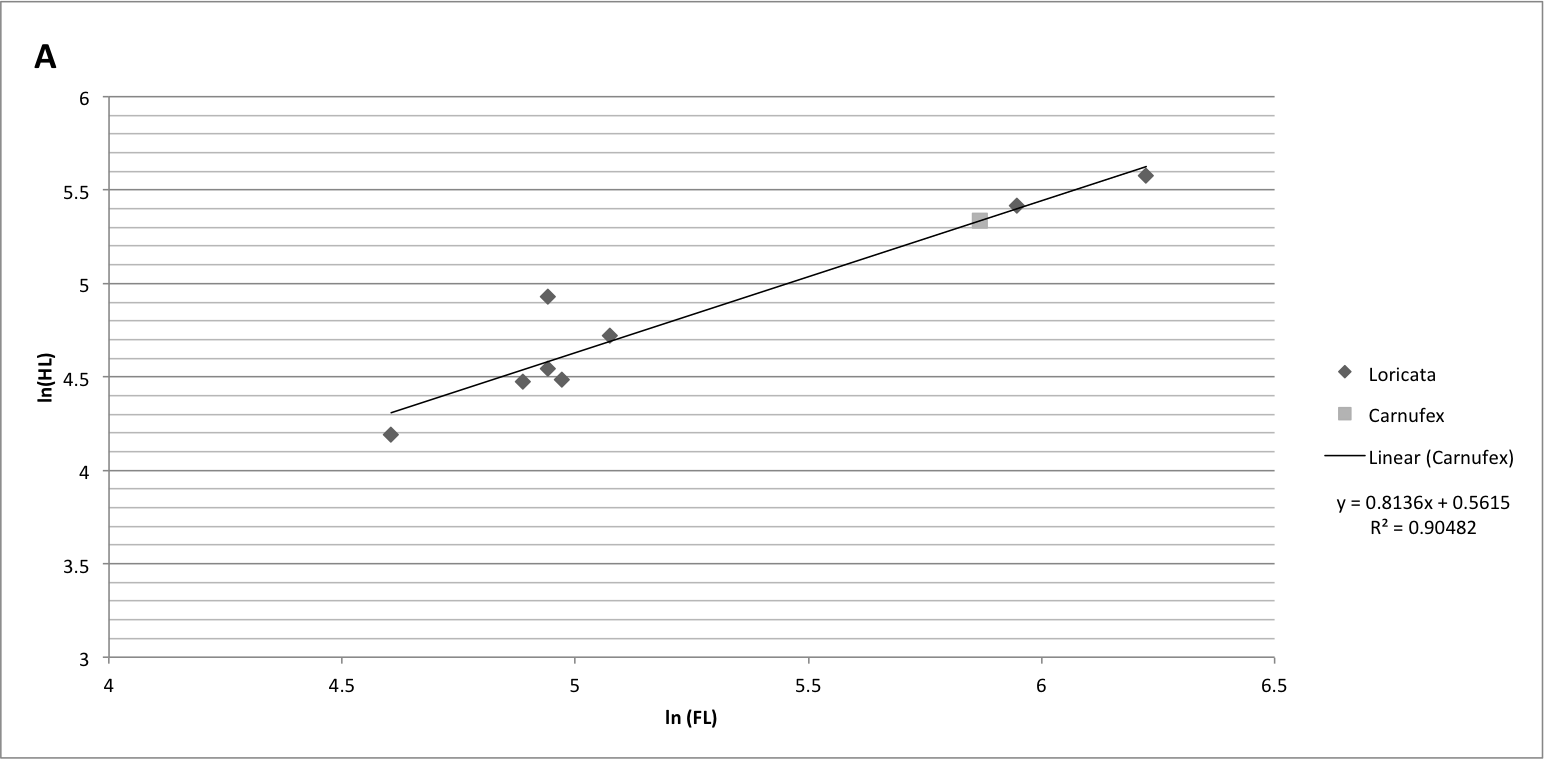


Figure S11. OLS linear regression of humeral length against femur length in Loricata. Calculated values of *Carnufex* plotted for reference.


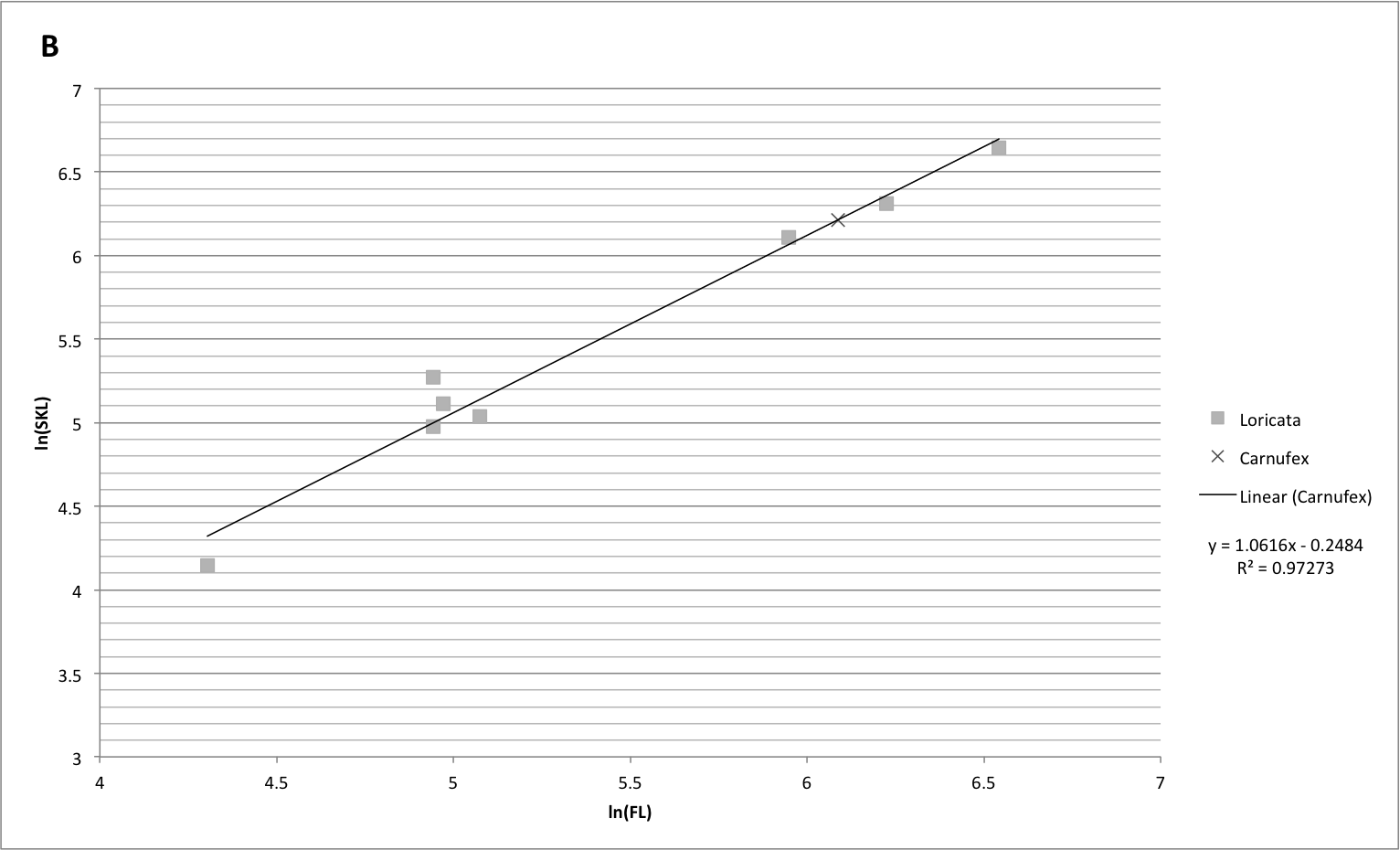


Figure S12. OLS linear regression of skull length against femur length in Loricata. Calculated values of *Carnufex* plotted for reference.


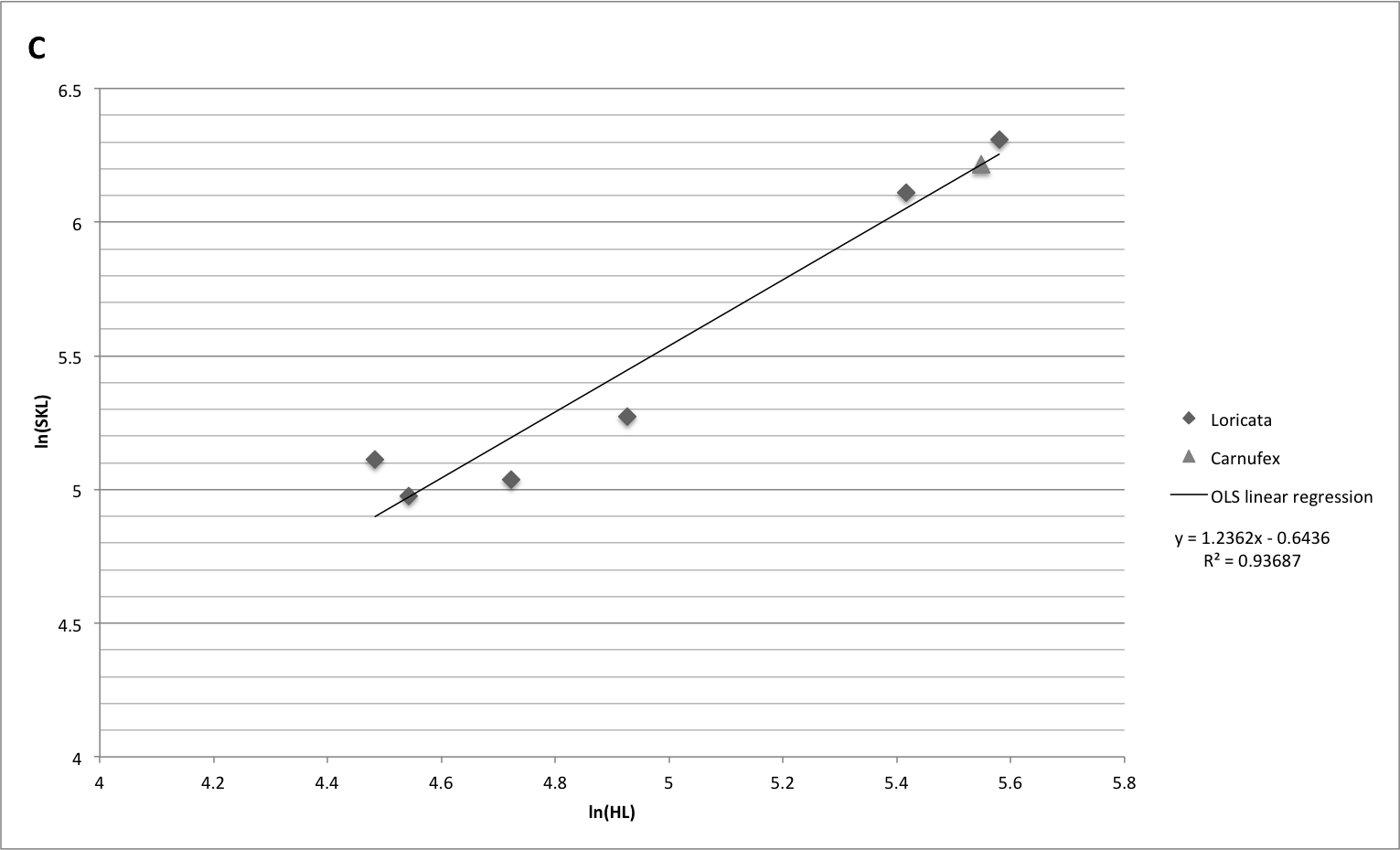


Figure S13. OLS linear regression of skull length against humeral length in Loricata. Calculated values of *Carnufex* plotted for reference.

| **Table S1.** **Taxonomic dataset for scaling equations.** Measurements in mm. | | | | | | | |
| --- | --- | --- | --- | --- | --- | --- | --- |
| **Taxon** | **FL** | **ln(FL)** | **HL** | **ln(HL)** | **SKL** | **ln(SKL)** | **Reference** |
| *Saurosuchus galilei* | 694 | 6.542 |  |  | 670 | 6.507 | Sill 1974 |
| *Postosuchus kirkpatricki* | 505 | 6.225 | 265 | 5.580 | 550 | 6.310 | Chatterjee 1985 |
| *Postosuchus kirkpatricki* | 383 | 5.948 | 225 | 5.416 | 450 | 6.109 | Chatterjee 1985 |
| *Hesperosuchus agilis* | 140 | 4.942 | 94 | 4.543 | 145 | 4.977 | Colbert 1952 |
| CM 29894 | 140 | 4.942 | 138 | 4.927 | 195 | 5.273 | Clark et al. 2000 |
| *Dromicosuchus grallator* | 144.3 | 4.972 | 88.6 | 4.484 | 166.3 | 5.114 | NCSM 13733 (UNC 15574) |
| *Dibothrosuchus elaphros* | 160 | 5.075 | 112.5 | 4.723 | 154 | 5.037 | Wu & Chattergee 1993 |
| *Litargosuchus leptorhynchus* | 74 | 4.304 |  |  | 63 | 4.143 | Clark & Sues 2002 |
| *Protosuchus haughtoni* | 90 | 4.500 |  |  |  |  | Turner & Nesbitt 2013 |
| *Protosuchus richardsoni* | 100 | 4.605 | 66 | 4.190 |  |  | Colbert & Mook 1951 |
| NCSM 21722 | 132.5 | 4.887 | 87.6 | 4.472 |  |  | NCSM 21722 |

| **Table S2. Femur length of carnivorous pseudosuchians used in bivariate plots of body mass and geologic time (Fig. 2B,C)**. Data sources listed. Measurements in mm. | | | | |
| --- | --- | --- | --- | --- |
| **Symbol (Fig. 2)** | **Taxon** | **FL** | **Reference** | **Age** |
| **CROCODYLOMORPHA** | | | | |
| **a** | *Carnufex carolinensis* | 353*-440* | NCSM 21558 | 231 Ma, Carnian, Late Triassic |
| **b** | *Dromicosuchus grallator* | 144 | Turner & Nesbitt 2013 | 226 Ma, early Norian, Late Triassic |
| **c** | CM 73372 | 290 | Turner & Nesbitt 2013 | Rhaetian, Late Triassic |
| **d** | *Redondavenator quayensis* | 523* | this paper | Rhaetian, Late Triassic |
| **e** | CM 29894 | 140 | Turner & Nesbitt 2013 | Rhaetian, Late Triassic |
| **f** | *Hesperosuchus agilis* | 140 | Colbert 1952 | early Norian, Late Triassic |
| **g** | *Terrestrisuchus gracilis* | 80 | Crush 1984 | Rhaetian, Late Triassic |
| **h** | *Saltoposuchus connectens* | 111 | Turner & Nesbitt 2013 | 215–212 Ma, Norian, Late Triassic |
| **i** | NCSM 21722 | 133 | NCSM 21722 | 231 Ma, Carnian, Late Triassic |
| **j** | *Trialestes romeri* | 203* | Reig 1962 | 229–231 Ma, Carnian, Late Triassic |
| **k** | *Pseudhesperosuchus* *jachaleri* | 159 | Turner & Nesbitt 2013 | 227–213 Ma, Norian, Late Triassic |
| **l** | *Kayentasuchus* *walker* | 129 | Turner & Nesbitt 2013 | Sinemurian, Early Jurassic |
| **m** | *Sphenosuchus* *acutus* | 140 | Turner & Nesbitt 2013 | Hettangian, Early Jurassic |
| **n** | *Litargosuchus* *leptorhynchus* | 74 | Turner & Nesbitt 2013 | Hettangian, Early Jurassic |
| **o** | *Pedeticosaurus* *leviseuri* | 113* | Walker 1970 | Sinemurian, Early Jurassic |
| **p** | *Dibrothosuchus* *elaphros* | 154*-167* | Wu and Chatterjee 1993 | Sinemurian, Early Jurassic |
| **q** | *Phyllodontosuchus* *lufengensis* | 71* | Harris et al. 2000 | Sinemurian, Early Jurassic |
| **RAUISUCHIDAE** | | | | |
| **A** | *Rauisuchus tiradentes* | 338 | Turner & Nesbitt 2013 | Carnian, Late Triassic |
| **B** | *Polonosuchus silesiacus* | 355 | Turner & Nesbitt 2013 | Carnian, Late Triassic |
| **C** | *Postosuchus kirkpatricki* | 528 | Long & Murry 1995 | early Norian, Late Triassic |
| **D** | *Postosuchus alisonae* | 558 | Turner & Nesbitt 2013 | 226 Ma, early Norian, Late Triassic |
| **BASAL LORICATA** | | | | |
| **E** | *Fasolasuchus tenax* | 750 | Turner & Nesbitt 2013 | late Norian, Late Triassic |
| **F** | *Batrachotomus kupferzellensis* | 420 | Turner & Nesbitt 2013 | Ladinian, Middle Triassic |
| **G** | *Saurosuchus galilei* | 694 | Turner & Nesbitt 2013 | Carnian, Late Triassic |
| **H** | *Prestosuchus chiniquensis* | 538 | Turner & Nesbitt 2013 | Ladinian, Middle Triassic |
| **POPOSAUROIDEA** | | | | |
| **I** | *Ticinosuchus ferox* | 240 | Krebs 1965 | Anisian, Middle Triassic |
| **J** | *Qianosuchus mixtus* | 491 | Li et al. 2006 | Anisian, Middle Triassic |
| **K** | *Arizonasaurus babbitti* | 490 | Turner & Nesbitt 2013 | Anisian, Middle Triassic |
| **L** | *Xilousuchus sapingensis* | 302 | Turner & Nesbitt 2013 | Olenekian, Early Triassic |
| **M** | *Poposaurus gracilis* | 353 | Long & Murry 1995 | Carnian–early Norian, Late Triassic |
| **N** | *Lotosaurus adentus* | 266 | Turner & Nesbitt 2013 | Anisian, Middle Triassic |
| **O** | *Sillosuchus longicervix* | 540 | Turner & Nesbitt 2013 | 229–231 Ma, Carnian, Late Triassic |
| **P** | *Shuvosaurus inexpectatus* | 255 | Turner & Nesbitt 2013 | early Norian, Late Triassic |
| **Q** | *Effigia okeeffeae* | 301 | Nesbitt 2007 | Rhaetian, Late Triassic |
| **GRACILISUCHIDAE** | | | | |
|  |  |  |  |  |
| **R** | *Gracilisuchus stipanicicorum* | 79.93 | Lecuona and Desojo, 2011 | Ladinian, Middle Triassic |
| **S** | *Yonghesuchus sangbiensis* | 146* | Butler et al. 2014 | 235–239 Ma, Ladinian, Middle Triassic |
| **T** | *Turfanosuchus dabanensis* | 135 | Wu and Russell 2001 | Ladinian, Middle Triassic |
| **ORNITHOSUCHIDAE** | | | | |
|  |  |  |  |  |
| **U** | *Riojasuchus tenuisceps* | 171 | Turner & Nesbitt 2013 | 227–213 Ma, Norian, Late Triassic |
| **V** | *Ornithosuchus longidens* | 87 | Colbert 1952 | Carnian–early Norian, Late Triassic |
| **W** | *Venaticosuchus rusconii* | 171* | Baczko & Ezcurra 2013 | 232–225 Ma, late Carnian-early Nornian |
| **DINOSAURIA** | | | | |
| **1** | *Staurikosaurus pricei* | 220 | Carrano 2006 | Carnian, Late Triassic |
| **2** | *Chindesaurus bryansmalli* | 265 | Long & Murry 1995 | 212 Ma, late Norian, Late Triassic |
| **3** | *Herrerasaurus ischigualastensis* | 482 | Carrano 2006 | 229–231 Ma, Carnian, Late Triassic |
| **4** | *Eoraptor lunensis* | 154 | Carrano 2006 | 229–231 Ma, Carnian, Late Triassic |
| **5** | *Guaibasaurus bandelariensis* | 214 | Carrano 2006 | early Norian Late Triassic |
| **6** | *Tawa hallae* | 174 | Irmis 2011 | 212 Ma, late Norian, Late Triassic |
| **7** | *Liliensternus liliensterni* | 424 | Carrano 2006 | late Norian, Late Triassic |
| **8** | *Procompognathus triassicus* | 92.5 | Fraas 1913 | 215–212 Ma, Norian, Late Triassic |
| **9** | *Coelophysis bauri* | 233 | Turner & Nesbitt 2013 | Rhaetian, Late Triassic |
| **10** | *Sanjuansaurus gordilloi* | 395 | Alcober & Martinez 2010 | 231 Ma, Carnian, Late Triassic |
| **11** | *Eodromaeus murphi* | 150.2 | Martinez et al. 2011 | 229–231 Ma, Carnian, Late Triassic |
| **12** | *Coelophysis* *rhodesiensis* | 203 | Carrano 2006 | Hettangian, Early Jurassic |
| **13** | *Cryolophosaurus* *ellioti* | 769 | Smith et al 2007 | Sinemurian-Pliensbachian, Early Jurassic |
| **14** | *Dilophosaurus* *wetherilli* | 552 | Carrano 2006 | Sinemurian, Early Jurassic |
| **15** | “*Dilophosaurus*” *sinensis* | 587 | Carrano 2006 | Hettangian, Early Jurassic |
| **16** | *“Syntarsus”* *kayentakatae* (MNA V2623) | 272 | Carrano 2006 | Sinemurian-Pliensbachian, Early-Middle Jurassic |
| **17** | *Daemonosaurus* *chauliodus* | 175* | Sues et al. 2011 | Rhaetian, Late Triassic |
| **18** | *Zupaysaurus rougieri* | 770* | Arcucci & Coria 2003 | Norian, Late Triassic |
|  |  |  |  |  |

| **Table S3. Select measurements of *Carnufex* *carolinensis* (NCSM 21623).** | | | |
| --- | --- | --- | --- |
| **Element** | **Measurement (mm)** | | |
| *Premaxilla* | alveolar margin length | 53 |  |
|  | posterior process length | 56 |  |
|  | total length | 90 |  |
| *External Naris* | length (min) | 20 |  |
| *Maxilla* | length (min) | 207 |  |
|  | height (min) | 108 |  |
| *Antorbital Fenestra* | length (min) | 154 |  |
|  | height (min) | 68 |  |
| *Lacrimal* | length | 84 |  |
|  | height | 89 |  |
| *Jugal* | length | 179 |  |
|  | height | 94 |  |
| *Orbit* | height (min) | 68 |  |
|  | width (min) | 50 |  |
| *Angular* | length | 328 |  |
| *Mandibular Fenestra* | length | 100 |  |
| *Articular* | length | 45 |  |
| *Humerus* | length | 210 |  |
|  | shaft width (long axis) | 22 |  |
|  | proximal end width | 71 |  |
|  | distal end width | 65 |  |
| *Vertebrae* |  | *Cervical* | *Dorsal* |
|  | centrum length (min) | 30 | 37 |
|  | neural spine height | 104 | 75 |
|  | total height | 146 | 103 |
