# Supplementary figures and images for: Early crocodylomorph increases top tier predator diversity during rise of dinosaurs
Source: Sci Rep. 2015 Mar 19;5:9276. doi: 10.1038/srep09276 (PMC4365386; doi:10.1038/srep09276)

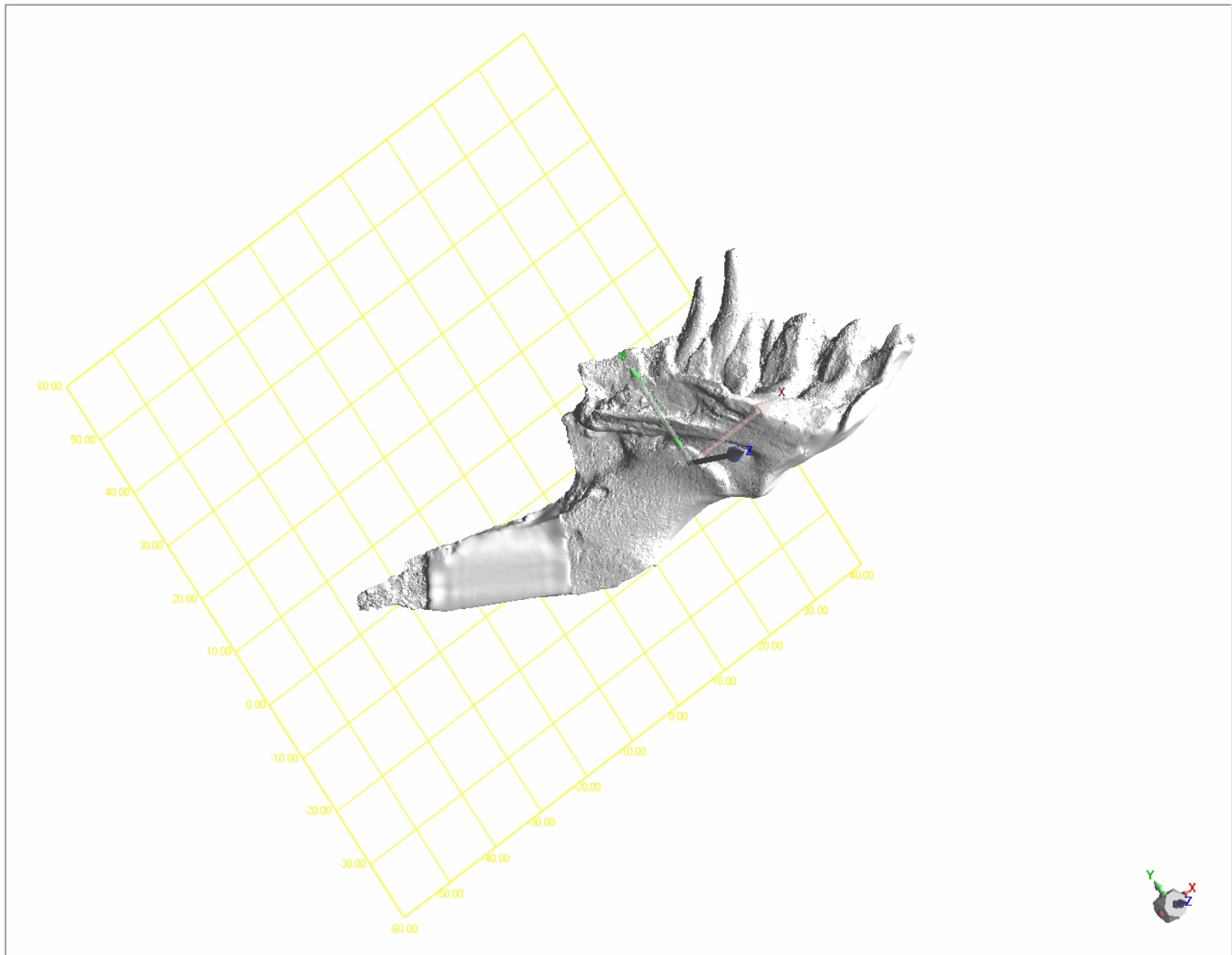

Click on the image to activate the 3D Model.

Supplement: Supplementary Information — Figure S1 [file srep09276-s2.pdf]

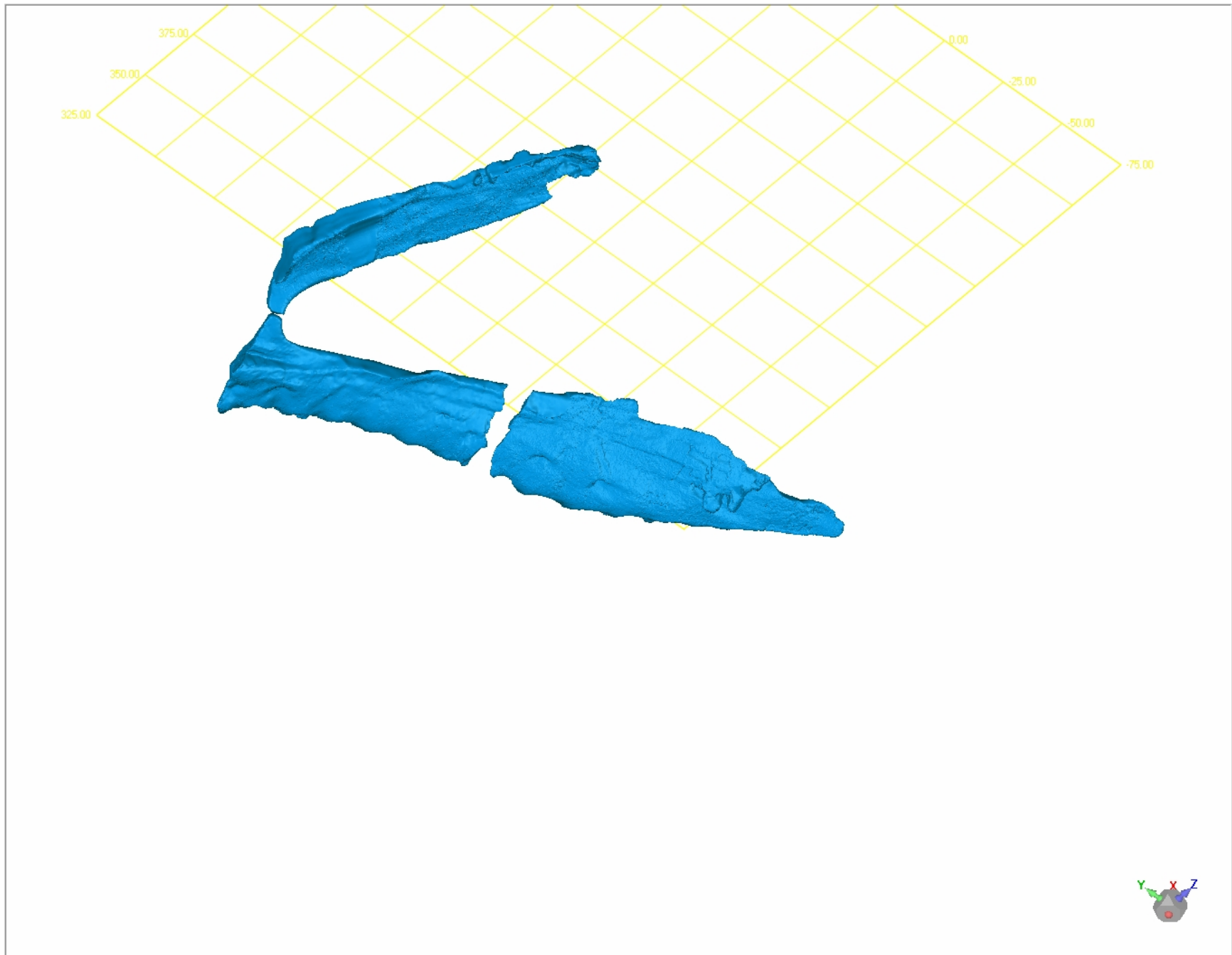

Click on the image to activate the 3D Model.

Supplement: Supplementary Information — Figure S2 [file srep09276-s3.pdf]

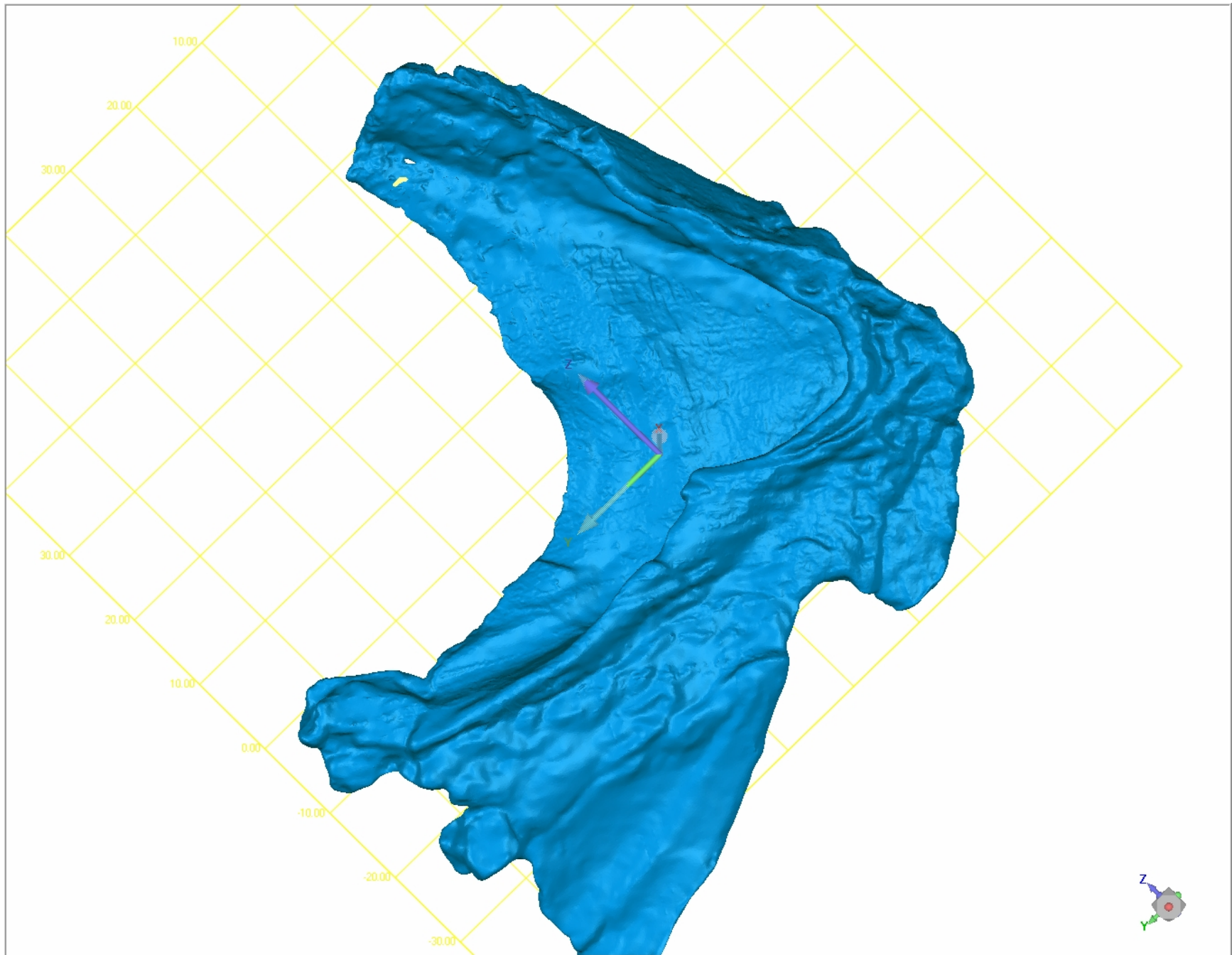

Click on the image to activate the 3D Model.

Supplement: Supplementary Information — Figure S3 [file srep09276-s4.pdf]

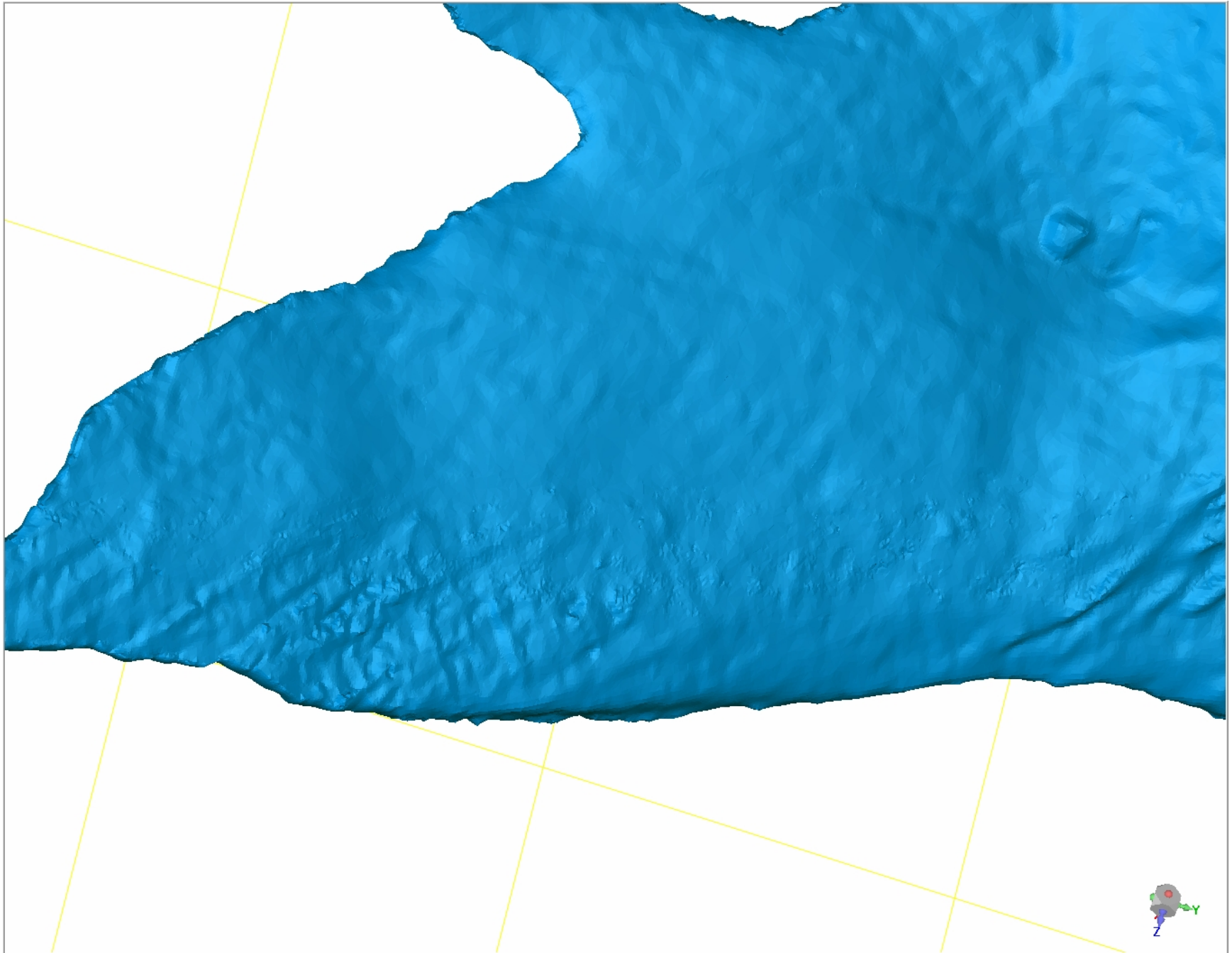

Click on the image to activate the 3D Model.

Supplement: Supplementary Information — Figure S4 [file srep09276-s5.pdf]

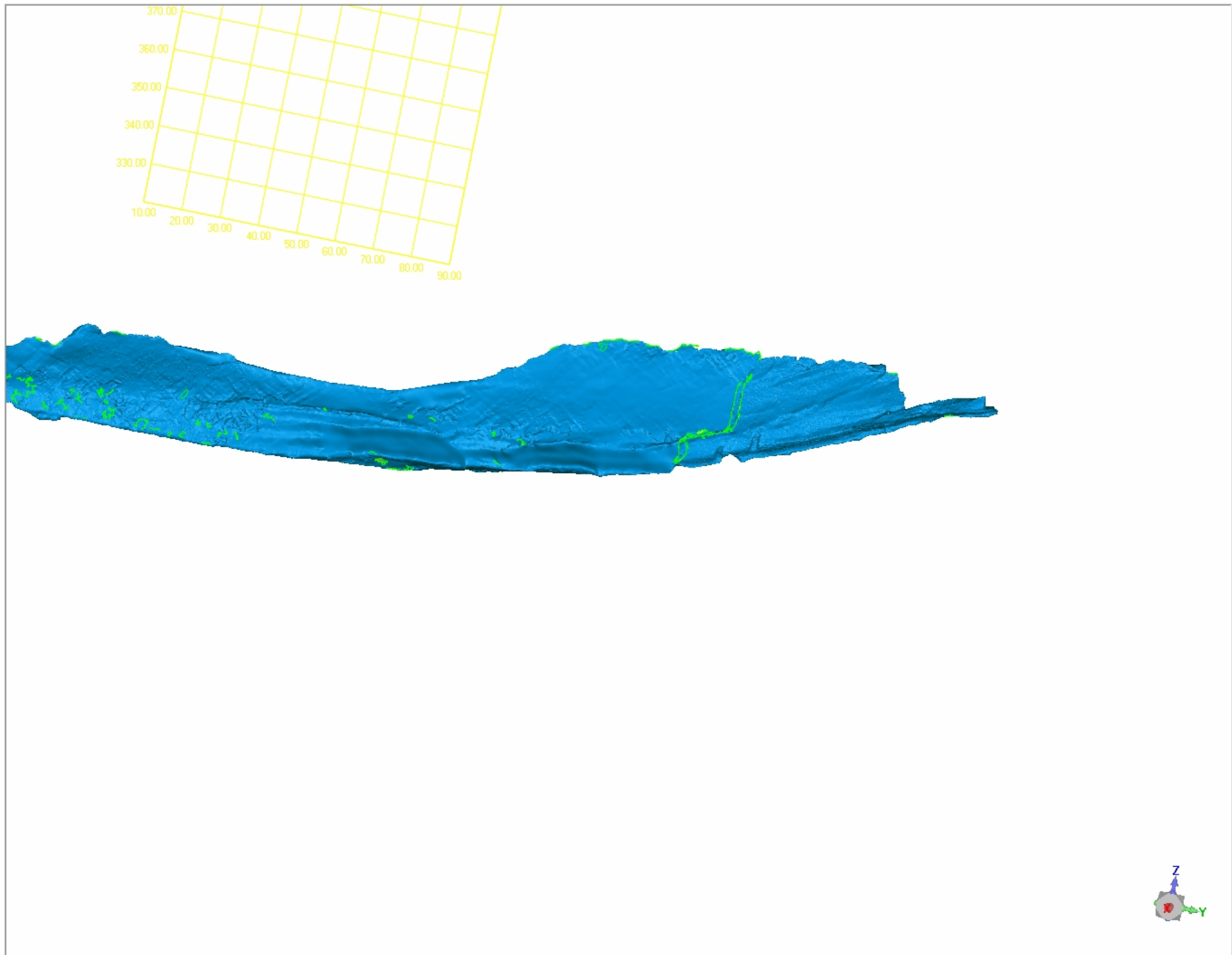

**Click on the image to activate the 3D Model.**

Supplement: Supplementary Information — Figure S5 [file srep09276-s6.pdf]

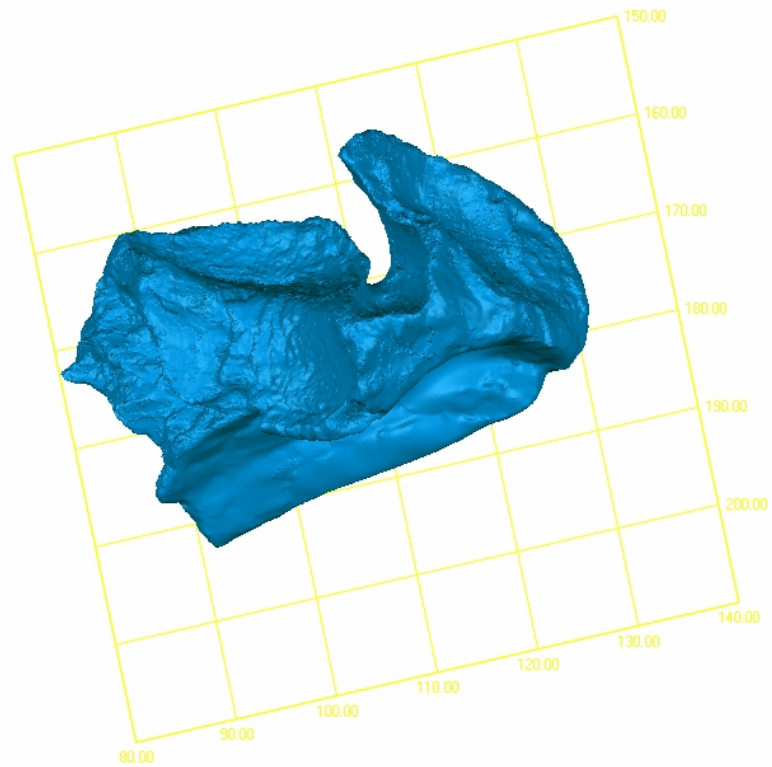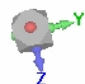

**Click on the image to activate the 3D Model.**

Supplement: Supplementary Information — Figure S6 [file srep09276-s7.pdf]

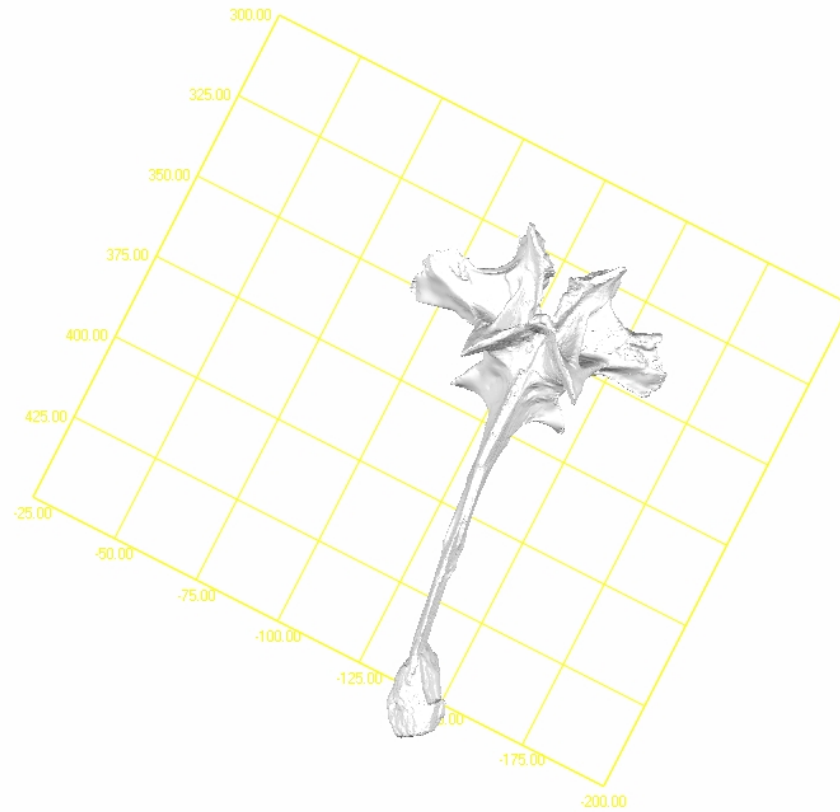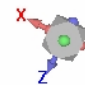

**Click on the image to activate the 3D Model.**

Supplement: Supplementary Information — Figure S7 [file srep09276-s8.pdf]

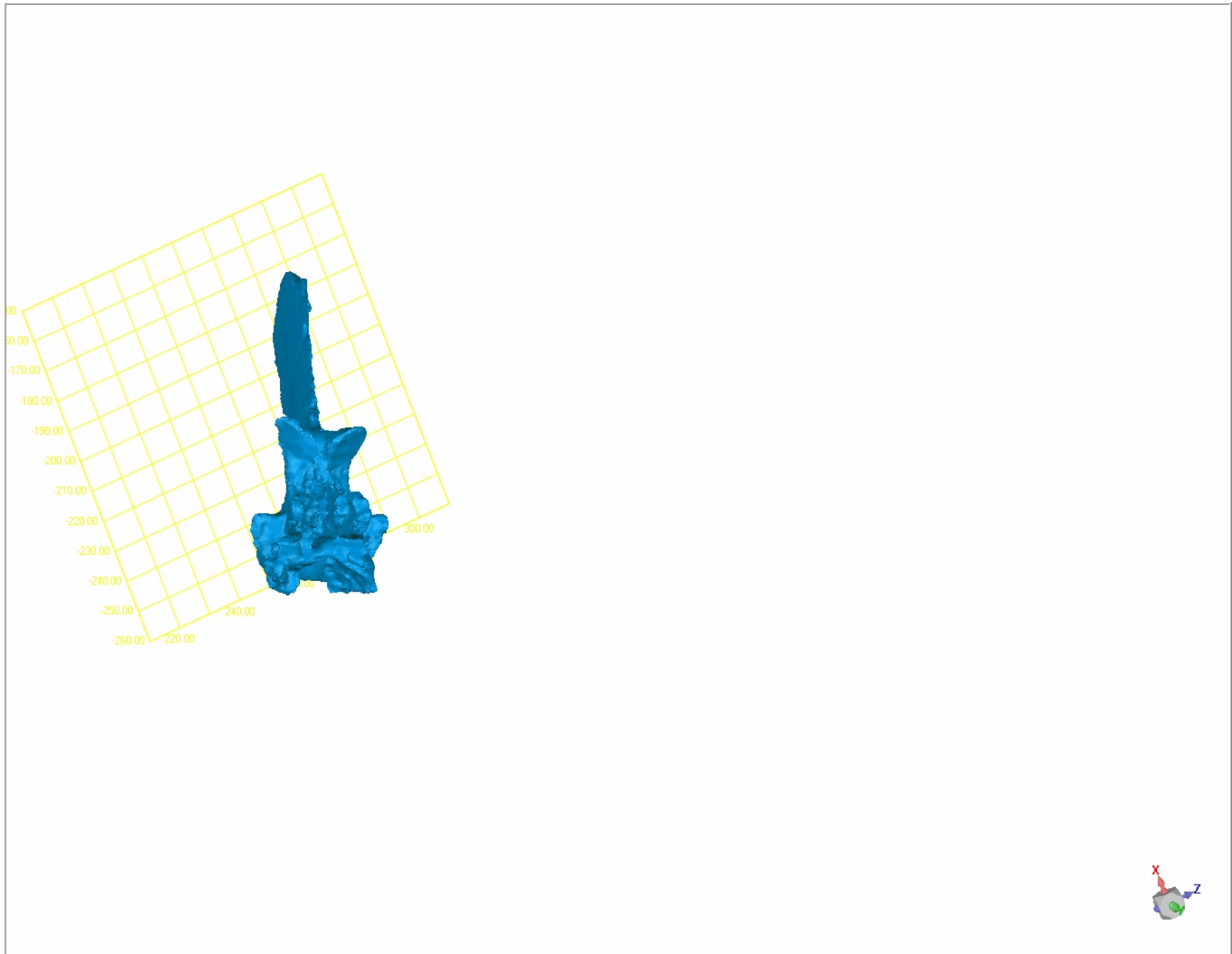

Click on the image to activate the 3D Model.

Supplement: Supplementary Information — Figure S8 [file srep09276-s9.pdf]
